# Sex-dependent effect of *APOE* on Alzheimer's disease and other age-related neurodegenerative disorders

**DOI:** 10.1242/dmm.045211

**Published:** 2020-08-27

**Authors:** Julia Gamache, Young Yun, Ornit Chiba-Falek

**Affiliations:** 1 Division of Translational Brain Sciences, Department of Neurology, Duke University Medical Center, Durham, NC 27710, USA; 2 Center for Genomic and Computational Biology, Duke University Medical Center, Durham, NC 27708, USA

**Keywords:** *APOE*, Late-onset, Alzheimer's disease, Sex effect, Neurodegenerative diseases, Gene-by-sex interaction

## Abstract

The importance of apolipoprotein E (*APOE*) in late-onset Alzheimer's disease (LOAD) has been firmly established, but the mechanisms through which it exerts its pathogenic effects remain elusive. In addition, the sex-dependent effects of *APOE* on LOAD risk and endophenotypes have yet to be explained. In this Review, we revisit the different aspects of APOE involvement in neurodegeneration and neurological diseases, with particular attention to sex differences in the contribution of *APOE* to LOAD susceptibility. We discuss the role of *APOE* in a broader range of age-related neurodegenerative diseases, and summarize the biological factors linking APOE to sex hormones, drawing on supportive findings from rodent models to identify major mechanistic themes underlying the exacerbation of LOAD-associated neurodegeneration and pathology in the female brain. Additionally, we list sex-by-genotype interactions identified across neurodegenerative diseases, proposing *APOE* variants as a shared etiology for sex differences in the manifestation of these diseases. Finally, we present recent advancements in ‘omics’ technologies, which provide a new platform for more in-depth investigations of how dysregulation of this gene affects the development and progression of neurodegenerative diseases. Collectively, the evidence summarized in this Review highlights the interplay between *APOE* and sex as a key factor in the etiology of LOAD and other age-related neurodegenerative diseases. We emphasize the importance of careful examination of sex as a contributing factor in studying the underpinning genetics of neurodegenerative diseases in general, but particularly for LOAD.

## Abstract (precis)

**Summary:** The *APOE ε4* allele and female sex are among the strongest risk factors for late-onset Alzheimer's disease. We explore how the interplay between these factors affects risk for this and other age-related neurodegenerative diseases.

## Introduction

Apolipoprotein E (*APOE*) was the first discovered and is by far the strongest genetic risk factor for late-onset Alzheimer's disease (LOAD). Since the discovery of the association between the *ε4* allele of *APOE* and LOAD risk (Roses, 1996; Strittmatter et al., 1993), the role of *APOE* and the protein it encodes, APOE, in disease pathogenesis has been rigorously investigated. Researchers discovered that *APOE ε4* exerts a stronger effect on disease risk in females than in males (Payami et al., 1994), and, over the years, the sex-specific effect of *APOE ε4* on LOAD has become extensively characterized. However, the molecular mechanisms underlying the role of *APOE ε4* in disease pathogenesis overall and at a sex-specific level remain unknown.

Recently, several key reports have put the spotlight on *APOE*. Foremost, recent studies described the protective effects of the Christchurch mutation (R136S) in the *APOE ε3* allele (Arboleda-Velasquez et al., 2019) and of *APOE ε2* homozygosity (Reiman et al., 2020). In addition, recent single-cell genomics studies identified LOAD-associated differential expression of *APOE* in specific subpopulations of glial cells (Grubman et al., 2019; Mathys et al., 2019; Nott et al., 2019). These findings not only reaffirm the importance of the *APOE* gene in LOAD, but also pinpoint its role in microglia as the potential culprit in disease pathogenesis. However, whether these effects are sex specific remains unexplored.

Here, we review the literature on *APOE*, with a focus on its understudied neurobiological functions and the sex-specificity of its pathogenic effect in LOAD and other neurodegenerative diseases. Although many of the articles we cite use the term ‘gender’ (see Glossary, Box 1), we will use the term ‘sex’ (Box 1) to emphasize the biological differences. We discuss various mechanisms based on accumulating evidence to explain the sex-dependent role of *APOE* in neurodegeneration.

**Box 1. Glossary**

**3xTgAD mice:** triple-transgenic mice harboring three human transgenes, each with mutations linked to familial Alzheimer's disease (AD) or frontotemporal dementia (*APP* KM670/671NL, *MAPT* P301L and *PSEN1* M146V).

**Amyloid-β** **(Aβ):** the oligomeric peptide formed by proteolytic cleavage of amyloid precursor protein (APP); Aβ aggregates form one of the pathological hallmarks of (LO)AD, amyloid plaques.

***APOE*-targeted replacement (*APOE*-TR) mice:** a humanized mouse model in which the murine *Apoe* gene is replaced with human *APOE ε2*, *ε3* or *ε4*; human *APOE* expression is controlled by murine regulatory sequences.

**APP/PS1 double-transgenic mice:** a mouse model harboring two human transgenes, each with mutations linked to familial AD (*APP* KM670/671NL and *PSEN1* L166P) and under the control of the neuron-specific *Thy1* minigene promoter.

**Blood-brain barrier:** a semi-permeable barrier between peripheral tissues and the central nervous system formed by a border of endothelial cells connected by tight junctions.

**Bulbar-onset** **ALS:** a form of amyotrophic lateral sclerosis (ALS) beginning with symptoms such as slurred speech and difficulty swallowing; involves upper motor neurons in the brain stem.

**Gender:** a broad description of sex that encompasses social and cultural differences; not discussed in this Review.

**J20 mice:** transgenic mice that overexpress human APP with two mutations linked to familial AD (*APP* KM670/671NL and *APP* V717F), driven by the *PDGFB* promoter.

**Hypothalamic-pituitary adrenal axis:** a stress response system linking the central nervous system with the endocrine system; composed of the hypothalamus, the pituitary gland and the adrenal gland.

**Incidence:** number of individuals developing a disease within a given time period, divided by total individuals at risk; represents new cases developing in a given time period.

**Life-long risk:** probability that an individual of a given age will develop a disease during their remaining life span.

**Limb-onset** **ALS:** a form of ALS in which symptoms such as muscle weakness and twitching begin in the legs and arms; involves lower motor neurons in the spinal cord; also called spinal-onset ALS.

**Long-term potentiation:** electrophysiological and structural strengthening of synapses due to patterns of activity; widely thought to underlie learning and memory.

**Neurotrophins:** a class of secreted growth factor proteins involved in cell survival, growth and differentiation; promote neuronal function.

**N-methyl-D-aspartate** **(NMDA) receptor** **(NMDAR):** an excitatory ionotropic receptor activated upon binding of either glutamate or glycine ligands.

**Polygenic risk score:** a calculation to predict disease risk based on known common genetic risk variants.

**PPAR-γ/PGC-1α pathway:** the signaling mechanism between PPAR-γ, a nuclear receptor, and PGC-1α, a transcriptional coactivator, that supports neuronal function by activating genes involved in energy metabolism.

**Prevalence:** number of individuals with a disease divided by total individuals in the population; represents the number of existing cases.

**Renin-angiotensin system:** a circulating hormone system that promotes blood pressure regulation and hydroelectrolytic balance; dysfunction is implicated in hypertension and cardiovascular risk.

**Sex:** either of two biological categories based on sex chromosome composition: male (XY) or female (XX).

**Tau:** the main axonal microtubule-associated protein in the mammalian central nervous system that forms one of the pathological hallmarks of (LO)AD, neurofibrillary tangles.

**Vascular dementia:** a broad term for cognitive dysfunction caused by impaired blood flow to the brain, usually due to multiple strokes.

## Sex differences in the epidemiology and manifestation of neurodegenerative diseases

Sex differences are established in the developing central nervous system during a period termed sexual differentiation, in which the brain is influenced by exposure to hormones and by changes in gene expression. These differences persist in the adult and aging brain, reflected in the neurological differences between healthy men and women. For example, synapse density is lower, whereas levels of tau (encoded by *MAPT*) and amyloid-β (Aβ; Box 1) are higher, in females than in males (Alonso-Nanclares et al., 2008; Buckley et al., 2019). Although women can be prone to developing neuropathology with aging, men are more likely to exhibit signs of neurodegeneration, especially in the hippocampus (Jack et al., 2017). However, once neurodegeneration begins in women, the rate of atrophy is faster than in men (Holland et al., 2013). These sex differences in how the healthy brain responds to the aging process may modulate susceptibility to developing neurodegenerative diseases.

### LOAD

Women are at higher risk of developing LOAD (Hebert et al., 2001), and the prevalence (Box 1) of LOAD is higher in females than in males (Prince et al., 2016). Out of 5.6 million people in the USA with LOAD aged 65 and older, 3.5 million were women and 2.1 million were men in 2019 (Alzheimer's Association, 2019). This sex bias has been attributed to increased longevity of females, as age is the biggest risk factor for LOAD. However, the incidence and life-long risk (Box 1) of LOAD remains higher in females even after adjusting for age and lifespan (Alzheimer's Association, 2016, 2019; Ruitenberg et al., 2001), suggesting that there are other biological causes for this discrepancy. Furthermore, women with LOAD exhibit worse cognitive decline and neuropathology than men with LOAD (Irvine et al., 2012). In fact, the rate of cognitive decline and brain atrophy is faster in women with mild cognitive impairment (MCI) and LOAD (Holland et al., 2013; Hua et al., 2010; Koran et al., 2017), and the impact of the neuropathology on this decline is greater in women (Barnes et al., 2005).

However, some studies have failed to detect sex differences in LOAD progression (Edland et al., 2002), perhaps due to aspects of study design, geographic region, statistical power and method of confirming LOAD diagnosis, such as with autopsy. Alarmingly, one meta-study found dramatic inconsistencies between clinical diagnoses and autopsy results, with less than 42% of participants across two studies showing alignment between LOAD pathology and diagnosis (Schneider et al., 2009). These inconsistencies, combined with a dearth of studies reporting findings stratified by sex (Fiest et al., 2016), present challenges for understanding whether women are truly more vulnerable to the development of LOAD. Nevertheless, there are compelling biological factors that generate hypotheses explaining why the female brain is more susceptible to this disease.

### Other neurodegenerative diseases

Sex differences have also been reported in the development and progression of multiple sclerosis (MS), Parkinson's disease (PD), dementia with Lewy bodies (DLB) and amyotrophic lateral sclerosis (ALS). MS is an autoimmune demyelinating disease with sex differences in prevalence, symptoms and severity. MS is more prevalent in women (Ramagopalan et al., 2010), who experience more frequent relapses and more inflammatory lesions (Kalincik et al., 2013; Pozzilli et al., 2003). Conversely, men with MS exhibit faster progression, exacerbated neurodegeneration and cognitive impairment, and greater cerebellar involvement (Antulov et al., 2009; Confavreux et al., 2003; Schoonheim et al., 2012).

Lewy body-spectrum diseases, including PD and DLB, also exhibit sex biases. PD is more prevalent in men than in women by a sex ratio of roughly 2:1 (Baldereschi et al., 2000). Similarly, DLB is more common in men (Savica et al., 2013). However, women with PD tend to exhibit increased mortality, dyskinesia, tremor and non-motor symptoms such as mood and sleep disturbances (Chandran et al., 2014; Dahodwala et al., 2016; Haaxma et al., 2007; Solla et al., 2012). Other aspects of PD, such as motor dysfunction and cognitive impairment, are more severe in men (Chandran et al., 2014; Lubomski et al., 2014; Szewczyk-Krolikowski et al., 2014).

Finally, ALS is a motor neuron disease that is more common and has an earlier age of onset in men than in women (Georgoulopoulou et al., 2011; McGuire et al., 1996). The clinical features of ALS are sex dependent, as women are more likely to develop bulbar-onset ALS (Box 1), whereas men are more likely to develop limb-onset ALS (Box 1) (Manjaly et al., 2010). Interestingly, a rare flail-arm presentation is much more common in men (Wijesekera et al., 2009), and men are also more likely to become cognitively impaired due to ALS (Portet et al., 2001). Biomarker studies suggest that ALS is more severe in men, but the disease-associated inflammatory response is exaggerated in women (Boylan et al., 2009; Nygren et al., 2002). In this Review, we propose the involvement of *APOE* polymorphisms in the manifestation of sex differences across these neurodegenerative diseases (Box 2).

**Box 2. *APOE* in shared sex-specific etiology across neurodegenerative diseases**

**Multiple sclerosis (MS)**

• The *APOE ε4* allele worsens MS severity in men (Table 1).

• *ε4*-associated APOE dysfunction is detrimental to myelin maintenance and repair (Bartzokis et al., 2006; Dean et al., 2014) and anti-inflammatory processes (Mailleux et al., 2017).

• Estrogen likely mediates a neuroprotective response in male brains, as local upregulation of aromatase and ERβ occurs near MS lesions in the male central nervous system (Luchetti et al., 2014), and genetic variance that reduces estrogen levels increases MS severity in men (Qureshi et al., 2017).

• Because APOE is required for some of the protective effects of estrogen, the *ε4* allele likely exacerbates disease severity in males via impaired function of APOE.

• Therefore, a combination of myelin impairment, neuroinflammation and loss of estrogen-mediated neuroprotection might contribute to *ε4*-associated MS severity in men.

**Lewy body spectrum diseases**

• *APOE* polymorphisms contribute to sex-specific risk for Parkinson's disease (PD) and dementia with Lewy bodies (DLB) (Table 1).

• The *APOE ε4* allele worsens α-synuclein pathology (Davis et al., 2020; Dickson et al., 2018; Zhao et al., 2020a) and cognitive function in PD (Mata et al., 2014).

• Dopamine transporter activity in the nigrostriatal dopaminergic system may underlie sex-specific vulnerability for developing PD (Gillies and McArthur, 2010), as males exhibit less efficient uptake and vesicular packaging of dopamine than females (Bhatt and Dluzen, 2005), possibly due to male-specific gene expression (Cantuti-Castelvetri et al., 2007) or renin-angiotensin system activity in the substantia nigra (Rodriguez-Perez et al., 2010).

• Estrogen attenuates the neurotoxic effects of 1-methyl-4-phenyl-1,2,3,6-tetrahydropyridine (MPTP), commonly used to induce PD in in animal models, on striatal dopamine release in female, but not male, rats (Disshon and Dluzen, 2000).

• Therefore, male dopaminergic neurons may be less responsive to the protective effects of estrogen, an effect exacerbated by lower plasma estradiol levels observed in male PD patients (Nitkowska et al., 2015).

• Collectively, these findings suggest that male dopaminergic neurons are more vulnerable to the pathogenic effects of the APOE ε4 isoform, making PD more prevalent and severe in men than in women.

**Amyotrophic lateral sclerosis (ALS)**

• The *APOE ε4* allele increases the risk for bulbar-, but not limb-onset, ALS in males (Table 1).

• Androgens have toxic effects on motor neurons (Katsuno et al., 2002); however, motor neuron atrophy can also arise from androgen insensitivity due to reduced androgen receptor (AR) expression (Grunseich et al., 2014).

• Because androgens can reverse some of the toxic effects of the ε4 isoform (Raber et al., 2002), ε*4-*positive males may be protected against limb-onset ALS due to the increased abundance of ARs in spinal motor neurons compared to other populations of motor neurons (Lumbroso et al., 1996; Sar and Stumpf, 1977).

• More sex-specific genetic polymorphisms associated with ALS continue to be discovered (Dunckley et al., 2007), warranting further research in this area.

• Of note, there is considerable clinical and genetic overlap between ALS and a related group of neurodegenerative diseases known as frontotemporal dementia, particularly in the development of TDP-43 (TARDBP) pathology (Boeve et al., 2012). Although *APOE ε4* was observed to be a risk factor for TDP-43 pathology in LOAD (Wennberg et al., 2018), whether or not this applies to ALS and frontotemporal dementia or has a sex-specific role remains to be determined.

**Table 1.**
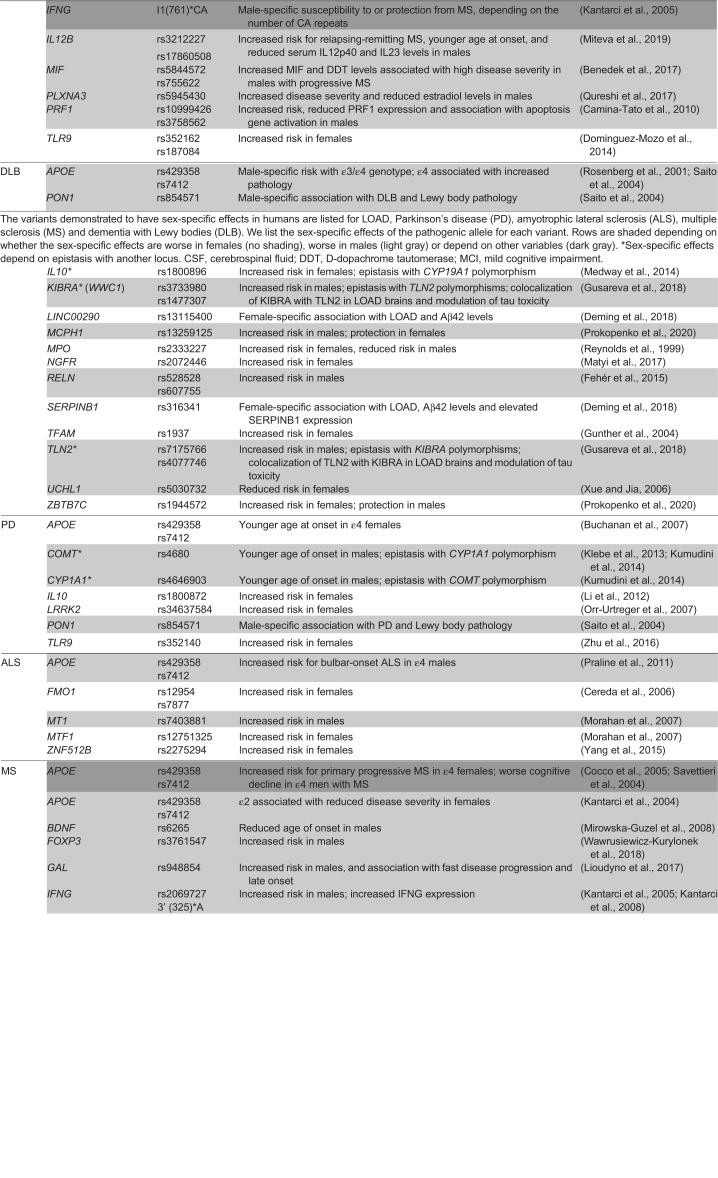
**Sex-by-genotype interactions across neurodegenerative disorders**

## Non-genetic sex-dependent determinants in LOAD

### Vascular and metabolic factors

Cardiovascular and metabolic conditions that develop in mid-life are risk factors for neurodegenerative diseases and increase the likelihood of cognitive dysfunction in late life (Kritz-Silverstein et al., 2017). The interactions of these conditions with risk for neurodegenerative disease is different between men and women. Men tend to have more coronary artery disease and cardiac arrhythmias, which correlate with LOAD biomarkers (Vemuri et al., 2017). This could explain the increased risk for vascular dementia (Box 1) in males (Ruitenberg et al., 2001), hypothesized to be attributed to a reduced activity of enzymes in the renin-angiotensin system (Box 1) specifically in males (Fernandez-Atucha et al., 2017). Interestingly, although hypertension is more common in middle-aged men, it is only a risk factor for dementia in women (Gilsanz et al., 2017) and is only associated with reduced cortical thickness in women (Kim et al., 2019). Women with poor metabolic phenotypes and high blood pressure have impaired cognitive performance compared to healthy post-menopausal women (Rettberg et al., 2016). In addition, men who survive to advanced age tend to have healthier cardiometabolic profiles overall, a survival bias that may contribute to the finding that women are at increased risk for neurodegenerative disease (Chêne et al., 2015).

### Inflammation

Generally, high levels of inflammation caused by vascular and metabolic disorders can increase one's risk for neurodegenerative diseases. Females are known to have a more robust peripheral immune response than males, which might relate to increased susceptibility to autoimmune disease. Studies showing sex differences in microglia, the immune cells of the brain, during neurodevelopment suggest that perturbation of immune pathways during early life stages may increase inflammation levels later in life and therefore increase the risk for neurodegenerative diseases. The proliferation and morphology of microglia in the developing mammalian brain is sex dependent, with males exhibiting greater densities of microglia, especially those of amoeboid morphology (Lenz et al., 2013; Schwarz et al., 2012). Acute immune activation with lipopolysaccharide accelerates the development of hippocampal microglia derived from male, but not female, wild-type mice (Hanamsagar et al., 2017), suggesting that higher sensitivity of the male brain to inflammation may accelerate microglial aging, affecting vulnerability to neurodegenerative disease. Indeed, sex differences found in developing microglia persist into adulthood, suggesting that sex-dependent microglial traits are determined at birth. In adult mice, male microglia display a pro-inflammatory bias, whereas female microglia express more genes involved in protection and repair mechanisms (Guneykaya et al., 2018; Villa et al., 2018). In support of the latter, female microglia respond more efficiently than male microglia to brain injury (Villa et al., 2018). In contrast, males display increased variability of microglial-specific gene expression with aging (Mangold et al., 2017), which might contribute to the development of neurological diseases. There is undoubtedly a relationship between the immune system and the development of LOAD, as most risk genes for this disease, including *CLU*, *TREM2*, *MS4A6A*, *CD33*, *PICALM* and *ABCA7* (Jansen et al., 2019; Kunkle et al., 2019), encode proteins related to immune functions. Whether or not the functional impact of these genetic variants differs between men and women, at the genomic or cellular level, remains to be studied.

### Sex hormones

Changes in hormone levels with aging can increase susceptibility to neurodegenerative diseases, as the human brain responds to estrogen, androgen and progesterone throughout life. Estrogen receptors are widely expressed in the brain, including in regions involved in learning and memory (Hutson et al., 2019), leading to the activation of both genomic and non-genomic pathways to regulate a range of neurobiological processes. Genomic pathways are mediated by the translocation of estrogen receptors (ERα and ERβ) to the nucleus upon estrogen binding (Kocanova et al., 2010). These estrogen-bound receptors can bind to estrogen response element motifs in chromatin (Palaniappan et al., 2019), transcription factors (Teyssier et al., 2001) or other coactivators (Shang et al., 2000) to modulate gene expression. The genes affected by estrogen treatment are involved in glucose transport and metabolism, mitochondrial function, inflammation, cholesterol homeostasis and myelination (Sárvári et al., 2011; Voskuhl et al., 2019; Zhao et al., 2012).

Aside from regulating gene expression, estrogen signaling directly affects the electrophysiological properties of neurons (Zhang et al., 2010), regulates long-term potentiation (Box 1) (Mukai et al., 2010) and promotes the activation of proteins with known roles in synaptic plasticity such as PSD-95 (DLG4) (Spencer-Segal et al., 2012). As estrogen receptors are located on axons, they are ideally positioned to regulate plasticity, and this axonal localization is more pronounced in female compared to male mice (Waters et al., 2015). The effects of estrogen on neuronal plasticity extend to structural factors, affecting the density and morphology of dendritic spines of hippocampal neurons (Mukai et al., 2010). Estrogen mitigates cellular stress by counteracting the production of reactive oxygen species and oxidative damage (Kemper et al., 2014; Numakawa et al., 2011), and has established anti-inflammatory properties, including reduction of microglial pro-inflammatory cytokine secretion (Habib et al., 2013). Finally, estrogen has been labeled as a master regulator of brain bioenergetics, helping to meet the high energy demands of neurons by increasing mitochondrial biogenesis and energy production via key elements in the tricarboxylic acid cycle, oxidative phosphorylation, respiratory efficiency and ATP synthesis (Kemper et al., 2014; Nilsen et al., 2007).

The irregular fluctuations in estrogen during the perimenopausal transition, as well as the dramatic loss of estrogen post-menopause, may make the female brain vulnerable to pathology and neurodegeneration via an ovarian-neural estrogen axis. In support of this, early surgical menopause doubles dementia risk, LOAD pathology and cognitive decline (Bove et al., 2014). In addition, loss of estradiol leads to hippocampal dysfunction (Jacobs et al., 2016; Rentz et al., 2017), and a reduced lifelong exposure to estrogen caused by delayed menarche and early menopause is associated with increased dementia risk (Gilsanz et al., 2019). Post-menopausal women with LOAD exhibit dysregulated alternative splicing of estrogen receptors, lower brain levels of estrogen and reduced estrogen sensitivity (Ishunina and Swaab, 2012; Rosario et al., 2011; Weiss et al., 2004), exacerbating the effect. In one study, post-menopausal women receiving hormone replacement therapy had reduced mortality due to LOAD and dementia (Manson et al., 2017). However, other reports suggest that hormone replacement therapy actually worsens cognitive decline, as discussed in a recent review (Marongiu, 2019).

Perimenopause coincides with the prodromal phase of LOAD, about 20 years before clinical diagnosis, a phase during which the pathological hallmarks of the disease, such as gliosis, begin to manifest (Jack et al., 2013). Increased indicators of LOAD endophenotypes, including hypometabolism, Aβ deposition and reductions in brain volume also begin at perimenopause (Mosconi et al., 2017a,b). Conditions like sleep disturbances and depression, which can arise during perimenopause, are associated with LOAD-like cognitive dysfunction and LOAD risk (Freeman et al., 2009; Hahn et al., 2014; Norton et al., 2014). During perimenopause, the female brain enters a hypometabolic state (Brinton et al., 2015), triggering a switch in energy source from glucose to ketone bodies (Yao et al., 2010), which are primarily derived from white matter (Klosinski et al., 2015). Perhaps due to this ketogenic switch, females, but not males, with LOAD and cerebrovascular disease exhibit dysregulation of myelin basic protein, a regulator of myelination (Gallart-Palau et al., 2016). Taken together, these findings suggest that menopause and the associated glucose hypometabolism are key players in determining female-specific susceptibility to LOAD. Because of the highly variable trajectory of hormone decline in women (Tepper et al., 2012), well-controlled research that includes reliably diagnosed perimenopause is needed to establish whether estrogen irregularities versus declines underlie the female-specific LOAD risk.

Similar to estrogen, testosterone has neuroprotective effects in brain; for example, against oxidative stress, neuronal injury and apoptosis through androgen receptors (Ahlbom et al., 2001; Hammond et al., 2001; Pike, 2001). Testosterone promotes neuronal growth (Brannvall et al., 2005), neurite outgrowth (Marron et al., 2005) and hippocampal long-term potentiation (Schulz and Korz, 2010). Consistent with these findings, reductions in androgens compromise brain function. Similar to menopause in women, men undergo a process known as andropause or androgen deficiency in aging males. This is more gradual than menopause and is not necessarily coupled to loss of reproductive function, but rather to dysfunction of androgen-responsive tissues (Feldman et al., 2002; Muller et al., 2003). Andropause is associated with cognitive dysfunction in men (Cherrier et al., 2015) and with risk for neurodegenerative diseases (Rosario et al., 2011). Men with LOAD have even lower levels of testosterone than age-matched, cognitively healthy men, further emphasizing the effects of testosterone loss in old age (Rosario et al., 2011). Some conflicting findings show that androgens can actually be harmful by reducing neuron survival (Johnson et al., 2010), but this may depend on the specific cell population and type of cellular stress being studied (Cunningham et al., 2009).

Growing evidence indicates that the neuroprotection conferred by sex hormones can counteract some aspects of neurodegenerative disease. At a genomic level, estrogen globally upregulates genes found to be downregulated in LOAD, especially those related to synaptic and mitochondrial function (Ratnakumar et al., 2019). In addition, testosterone supplementation in older men modestly improves cognition (Wahjoepramono et al., 2016). Sex hormones have also been shown to be neuroprotective against Aβ and tau toxicity (Park et al., 2007; Pike, 2001; Wang et al., 2016), as well as the mitochondrial deficits associated with these pathologies (Grimm et al., 2016).

## *APOE*: a sex-dependent genetic determinant in LOAD

**Fig. 1.**
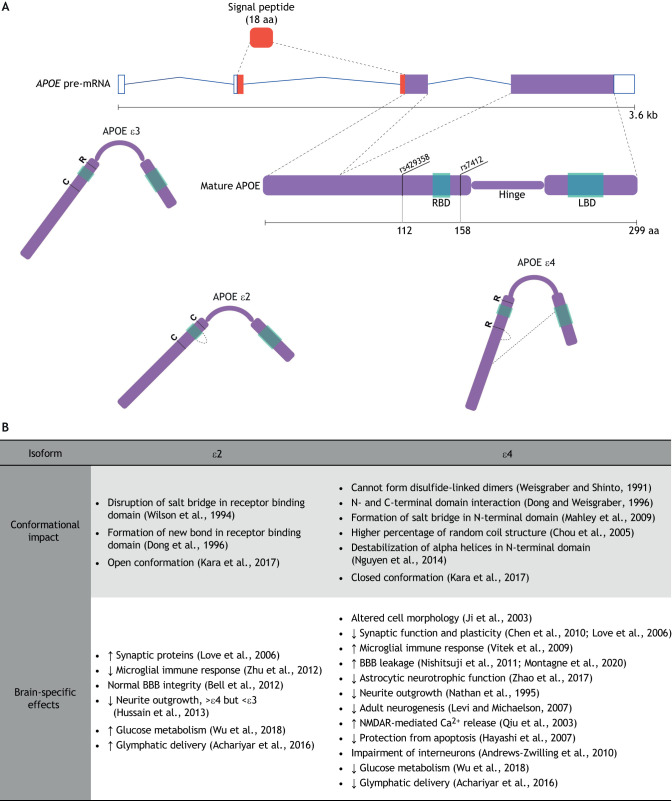
***APOE* variants differentially affect its conformational properties and biological effects in the brain.** (A) Diagram of *APOE* pre-mRNA, which consists of four exons to encode a 317-residue pre-APOE protein. An 18-residue signal peptide is co-translationally removed from pre-APOE (Lin-Lee et al., 1981; Zannis et al., 1984) to produce the mature 299-aa APOE, which consists of N-terminal and C-terminal arms connected by a hinge region (Wetterau et al., 1988). A receptor-binding domain (RBD) is located in the N-terminal arm (Wilson et al., 1991), while a lipid-binding domain (LBD) is located in the C-terminal arm (Weisgraber, 1990). Two single-nucleotide variants affect the aa sequence around the RBD – rs42958 (aa 112, C→R, ε4, 14% allele frequency) and rs7412 (aa 158, R→C, ε2, 7% allele frequency) (Bertram et al., 2007). These aa changes result in different APOE isoforms with altered functions. The schematic drawings of the three isoforms illustrate the shapes as described in B. (B) Table listing the effects of aa changes in the ε2 and ε4 isoforms of APOE relative to ε3, except where otherwise specified. aa, amino acids; BBB, blood-brain barrier; kb, kilobases; NMDAR, N-methyl-D-aspartate receptor.

*APOE* encodes three protein isoforms designated ε2, ε3 and ε4 based on two single-nucleotide variants that alter the amino acid sequence at positions 112 (rs429358, C/T) and 158 (rs7412, C/T) (Weisgraber, 1994) (Fig. 1A). The frequencies of the alleles in the global population are 7% for *ε2*, 79% for *ε3* and 14% for *ε4*, with the *ε3/3* (62%), *ε3/4* (22%) and *ε2/3* (11%) genotypes being the most common (Bertram et al., 2007). The amino acid differences at positions 112 and 158 affect protein conformation relative to the more common *ε3* allele (Fig. 1A), and studies show that these consequently affect APOE biology and its brain-specific functions (Fig. 1B). As outlined in Fig. 1B, unique domain interactions in the ε2 (Dong et al., 1996; Wilson et al., 1994) and ε4 (Dong and Weisgraber, 1996; Mahley et al., 2009) isoforms result in a more open or closed conformation, respectively, compared to ε3 (Kara et al., 2017). In addition, hydrophobic residue exposure is exaggerated in the ε4 isoform compared to ε3 (Chou et al., 2005), leading to structural instability, increased lipid-binding efficacy (Nguyen et al., 2014) and inability to dimerize (Weisgraber and Shinto, 1991). These contrasting conformational properties between APOE isoforms have consequences for synaptic function (Chen et al., 2010; Love et al., 2006; Qiu et al., 2003), glial cell function (Vitek et al., 2009; Zhu et al., 2012), blood-brain barrier (Box 1) integrity (Bell et al., 2012; Montagne et al., 2020; Nishitsuji et al., 2011), generation and differentiation of newborn neurons (Levi and Michaelson, 2007; Nathan et al., 1995), and distribution of APOE in the brain (Achariyar et al., 2016). The following section explores the multifunctional nature of APOE in the brain, explaining why differential effects of APOE isoforms extend to cell survival (Andrews-Zwilling et al., 2010; Hayashi et al., 2007; Zhao et al., 2017), glucose delivery to cells (Wu et al., 2018), formation of toxic fragments (Huang et al., 2001; Love et al., 2017) and gene expression patterns (Lin et al., 2018) (Fig. 1B).

### Brain-specific functions of APOE

A number of recent reviews discuss the structure and function of APOE (e.g. Abondio et al., 2019; Marais, 2019). However, as APOE biology in the brain is distinct from that in the periphery, a summary of its brain-relevant functions here provides helpful context for understanding subsequent discussions of its sex-specific role in LOAD. The glycoprotein APOE is coded from four exons on chromosome 19 (Fig. 1A). APOE is composed of an N-terminal arm that contains a receptor-binding domain, and a C-terminal arm, which contains a lipid-binding domain (Fig. 1A). To resolve the three-dimensional structure of monomeric APOE, a recent study combined cross-linking and mass spectrometry at high resolution to show that APOE is highly dynamic, adapting its conformation to dimerize and bind receptors and lipids (Henry et al., 2018). APOE is a lipid-transport protein expressed most highly in the liver and kidney, but also at relatively high levels in most areas of the brain (Fagerberg et al., 2014). In the brain, *APOE* is predominantly expressed in astrocytes and, to a lesser extent, in microglia and neurons (Xu et al., 2006), and the major APOE pathway in the brain is astrocyte derived. Naïve APOE secreted from astrocytes undergoes lipidation to form high-density lipoprotein (HDL) particles (Fagan et al., 1999). Although APOE has many interacting partners, it is mainly lipidated by the ATP-binding cassette transporter ABCA1 (Kim et al., 2007). Lipid-rich APOE then interacts predominantly with low-density lipoprotein (LDL) receptors (LDLRs) on microglia and neurons, facilitating the endocytosis of cholesterol and phospholipids. Single-cell genomics studies align with growing evidence indicating cell type-specific functions of APOE (Box 3).

**Box 3. *APOE* and single-cell genomics studies of LOAD**

The brain is a highly heterogeneous tissue composed of many different cell types. The notion of cell type-specific responses to LOAD progression is not novel – it has been firmly established that neurons are lost, whereas glial cells proliferate. Multiple lines of evidence suggest that the function of APOE is cell type specific. For instance, while most astrocytes express APOE, a subset do not, and a subset of microglia do not increase APOE production in response to injury (Xu et al., 2006). In addition, APOE from specific cell types has stronger affinities for specific receptors; for instance, astrocyte-derived APOE has a strong affinity for LDLR (Fryer et al., 2005). LRP1 is mainly found on neurons, whereas LDLR is more prominently expressed in glia (Rapp et al., 2006), possibly inducing cell type-specific downstream effects of APOE. Advanced single-cell technologies now allow researchers to interrogate cell subtype-specific genomic profiles with unprecedented cellular resolution and are becoming more widespread. Two recent single-cell studies of human brain tissue derived from LOAD and healthy control subjects characterized the cell type-specific pathogenic mechanisms of LOAD and revealed that APOE is upregulated in a disease-associated subpopulation of microglia, whereas it is downregulated in oligodendrocyte and astrocyte subpopulations (Grubman et al., 2019; Mathys et al., 2019). These findings are highly significant for our understanding of APOE function and dysfunction in the brain, and may open the door for novel therapeutic strategies such as genome editing systems that target specific cell subpopulations.

APOE redistributes lipids between cells, modulating lipid homeostasis in the brain. Lipids are central to the structure and function of all cells, as they are the principal components of cell membranes, contributing to membrane fluidity and remodeling. There is a particularly high demand for lipids such as cholesterol in the brain, which contains up to 25% of the body's total cholesterol (Bjorkhem and Meaney, 2004). Although there are other cholesterol transporters throughout the body, APOE is by far the most abundant in the brain. As a regulator of brain cholesterol homeostasis, APOE plays a role in many of the same functions that require cholesterol, including neurite outgrowth in cultured olfactory epithelia (Hussain et al., 2013), synapse formation in cultured retinal ganglion cells (Mauch et al., 2001), maintenance of myelin as shown by diffusion tensor imaging of healthy human adults (Westlye et al., 2012), synaptic integrity and plasticity as demonstrated by *Apoe* knockout mice (Lane-Donovan et al., 2016), and dendritic complexity of neurons in both *APOE*-targeted replacement (*APOE*-TR) mice (Box 1) and human LOAD patients (Ji et al., 2003). APOE also helps maintain the structural integrity of the blood-brain barrier in immune-challenged wild-type compared to *Apoe* knockout mice (Zheng et al., 2014), regulates the development of newborn neurons during adult neurogenesis, as shown by comparing wild-type, *Apoe* knockout and *APOE*-TR mice (Tensaouti et al., 2018), and helps remove debris from degenerating cells in wild-type mice with induced demyelinating lesions in the brain (Cantuti-Castelvetri et al., 2018). The involvement of APOE in such a diverse array of functions adds layers of complexity to understanding its biological function in the brain and in neurodegenerative diseases.

### APOE and sex hormones

APOE is regulated by estrogen, progesterone and testosterone, raising the possibility that hormone-APOE interactions underlie the sex differences in susceptibility to neurodegenerative disease and, furthermore, that the role of APOE in LOAD pathogenesis is sex specific. Estrogen increases the synthesis and expression of APOE in the brain (Ratnakumar et al., 2019; Zhao et al., 2012). Downstream effects of estrogen administration on APOE expression depend on whether ERα or ERβ is predominantly activated. Wang and colleagues used cultured rat hippocampal neurons and an *in vivo* rat model to show that *Apoe* mRNA and protein expression increase in response to ERα activation and decrease by ERβ activation (Wang et al., 2006); however, ERα can outcompete and displace ERβ from chromatin (Charn et al., 2010).

APOE facilitates the neuroprotective effects of estrogens and androgens. Estradiol-mediated increases in APOE result in neurite outgrowth in cultured mouse cortical neurons (Nathan et al., 2004), and in neuronal maturation and synaptogenesis in the murine olfactory bulb (Nathan et al., 2010). APOE is also required for estrogen-mediated neuroprotection in a mouse model of global ischemia (Horsburgh et al., 2002). In addition, studies suggest a link between estrogen-APOE neuroprotection and the hypothalamic-pituitary adrenal axis (Box 1). Cortisol/corticosterone exacerbates neurotoxicity (Goodman et al., 1996), and estrogen counteracts this via an ERα-corticotropin-releasing hormone-binding protein (CRH-BP) pathway (van de Stolpe et al., 2004). APOE may be involved in tonic inhibition of the hypothalamic-pituitary adrenal axis through estrogen signaling via reduction of cortisol levels (Raber et al., 2000). Testosterone has similar APOE-mediated anti-inflammatory effects to estrogen (Brown et al., 2007).

The literature on cholesterol metabolism and menopause provides further evidence for a mechanistic link between estrogen and APOE. Estrogen regulates elements of cholesterol synthesis and uptake, including LDLR-related protein 1 (LRP1), LDLR (Cheng et al., 2007) and hydroxymethylglutaryl-CoA reductase (HMG-CR) (De Marinis et al., 2008). HMG-CR transcription is activated by SREBP2, which is co-activated with APOE by cholesterol metabolism in astrocytes (Abildayeva et al., 2006). These studies suggest a potential convergence of estrogen and APOE signaling on the SREBP2/HMG-CR pathway to regulate cholesterol transport and homeostasis in the brain. This pathway may affect Aβ and tau pathology through LRP1 and cholesterol-ester mechanisms (van der Kant et al., 2019). In fact, APOE ε4-mediated Aβ pathology likely depends on LRP1, as LRP1 deficiency in neurons blocks the APOE ε4-mediated exacerbation of amyloid plaque burden in APP/PS1 (PSEN1) double-transgenic mice (Box 1) (Tachibana et al., 2019). Estrogen also reduces LDL cholesterol levels (Ghosh et al., 2015; Hussain et al., 2015); however, it remains unknown whether this affects APOE and/or lipid homeostasis in the brain. Finally, *APOE* genotype modulates estradiol levels and age at menopause – the *ε3*/*ε3* genotype is associated with later menopause and lower estradiol serum concentrations than the other five genotypes (Ryl et al., 2016), while another study found that the presence of at least one *ε2* allele is associated with later menopause than *ε3*/*ε3* (Tempfer et al., 2005). Early menopause is thought to be linked to increased LOAD risk due to reduced life-long exposure to estrogen (Gilsanz et al., 2019).

In conclusion, owing to the interactions of APOE and sex hormones through various pathways, fluctuations and/or loss of estrogens at perimenopause and post-menopause may contribute to the increased susceptibility to and severe manifestation of LOAD in females through APOE. The presence of the high-risk *APOE ε4* allele might further exacerbate this sex-specific effect.

### APOE ε4

It has been firmly established that the *APOE ε4* allele increases the risk for developing non-Mendelian AD (Roses, 1996; Strittmatter et al., 1993). *APOE ε4* is associated with earlier disease onset (Corder et al., 1993), heavier amyloid pathology (Jack et al., 2015; Schmechel et al., 1993), increased brain atrophy (Agosta et al., 2009; Tosun et al., 2010) and faster rate of disease progression (Stone et al., 2010). A multi-center meta-analysis genome-wide association study (GWAS) showed that of all genetic loci contributing to LOAD risk, the *APOE* linkage disequilibrium region is by far the strongest (Kunkle et al., 2019). This association was attributed, at least in part, to *APOE ε4*.

**Fig. 2.**
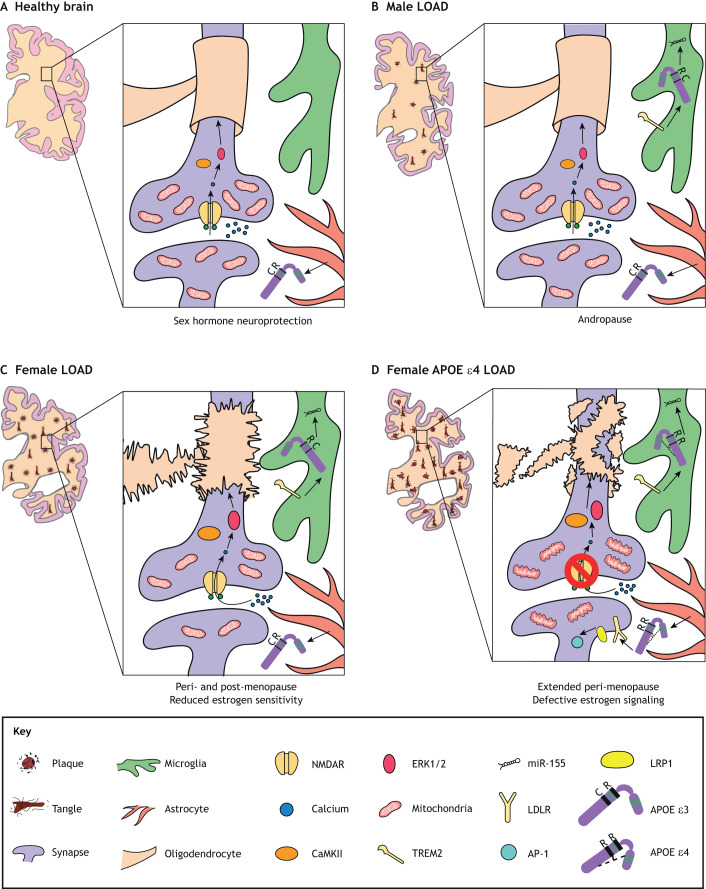
**Exacerbation of LOAD neurodegeneration and pathology by female sex and *APOE ε4*, and hypothesized mechanisms.** (A) The healthy *APOE ε3/ε3* brain benefits from neuroprotection conferred by sex hormones, with abundant mitochondria at synapses, intact myelin, intact NMDAR-mediated neuroplasticity and homeostatic microglia. (B) The male LOAD brain exhibits degeneration, neuropathology, reduced mitochondria and pro-inflammatory signature in microglia, in part due to the gradual loss of sex hormones during andropause. (C) With fluctuations and declines in sex hormones at peri- and post-menopause, in addition to reduced estrogen sensitivity, the female LOAD brain exhibits more severe atrophy and neuropathology, loss of mitochondria and myelin abnormalities. (D) *APOE ε4* is associated with defective estrogen signaling and a delay in menopause, which may lengthen the perimenopausal transition period of intense estrogen level fluctuations. Therefore, the female *APOE ε4*-positive LOAD brain exhibits the most severe atrophy and neuropathology, disruption of the myelin sheath, mitochondrial deficits, impairments in neuroplasticity and increased activation of AP-1, which drives APP expression and, consequently, Aβ deposition. LDLR, low-density lipoprotein receptor; LRP1, LDLR-related protein 1; NMDAR, N-methyl-D-aspartate receptor.

Studies have demonstrated a greater risk and earlier development of LOAD (Bretsky et al., 1999; Fisher et al., 2018; Poirier et al., 1993) and a higher MCI-LOAD conversion risk (Altmann et al., 2014) in *ε4*-carrier females than *ε4*-carrier males. Strikingly, one study found that heterozygote *ε4*/– women have the same level of risk as homozygote *ε4/ε4* men (Payami et al., 1994). In addition to increased risk for LOAD, female carriers of *APOE ε4* exhibit more severe presentation of endophenotypes including more severe Aβ and tau pathology (Corder et al., 2004), worse cognitive decline (Beydoun et al., 2012; Lin et al., 2015) and worse atrophy (Fleisher et al., 2005; Sampedro et al., 2015) (Fig. 2). Although the vast majority of studies on the relationship between *APOE ε4* and LOAD risk showed increased vulnerability in women compared to men, some reported conflicting findings (Fisher et al., 2018). However, these age-matched studies do not account for sex differences in age of diagnosis and disease trajectories, which are accelerated in females (Corder et al., 2004; Cosentino et al., 2008; Holland et al., 2013). The confounding effects of increased cardiovascular disease risk in men can also influence the results due to survival bias (Beydoun et al., 2013).

The mechanisms underlying this female-specific effect of the *APOE ε4* allele on LOAD risk are largely unknown, but studies suggest that the *APOE ε4* allele exacerbates the effects of fluctuations/loss of sex hormones during peri-/post-menopause. APOE ε4 negates the beneficial effects of estrogen on neurite extension (Nathan et al., 2004) and on anti-inflammatory responses in microglia (Brown et al., 2008). The *APOE ε4* allele can also have an additive effect on LOAD-associated brain glucose hypometabolism (Ossenkoppele et al., 2013; Reiman et al., 2005), as this allele contributes to glucose hypometabolism even in the absence of neurodegeneration and cognitive decline (Knopman et al., 2014; Sundermann et al., 2018). As the *ε4* allele is associated with delayed menopause (Meng et al., 2012), it perhaps lengthens the perimenopausal transition and worsens glucose hypometabolism. A proposed mechanism underlying *ε4*-associated hypometabolism is the inhibition of the PPAR-γ/PGC-1α (PPARGC1A) pathway (Box 1), which supports neuronal function by promoting mitochondrial energy production (Wu et al., 2018). APOE ε4 is thought to reduce PGC-1α activity via downregulation of the deacetylase sirtuin 1 (SIRT1) (Theendakara et al., 2016). Because estrogen counteracts this mechanism by upregulating SIRT1 (Theendakara et al., 2016), age-related estrogen loss in women may exacerbate ε4-induced brain hypometabolism. In line with the ketogenic switch that co-occurs with menopause and the fact that ketone bodies are primarily derived from white matter, LOAD and its associated endophenotypes are linked to reductions in white matter and dysregulation of myelination (Gallart-Palau et al., 2016; Mosconi et al., 2017b), and *ε4* homozygotes exhibit increased white matter diffusivity, most likely due to myelin sheath disruption (Operto et al., 2018). Overall, these findings support the idea that estrogen loss underlies the increased vulnerability to neurodegeneration in the *APOE ε4-*positive female brain via energy imbalance (Fig. 2).

The sex-specific effects of APOE ε4 can also occur at a genomic level. APOE ε4 acts as a transcription factor in the brain, binding the promoter regions of genes associated with programmed cell death, microtubule disassembly, synaptic function and aging (Theendakara et al., 2016). APOE ε4-mediated transcriptional activity was found to be sex specific, with genes involved in regulation of the immune system response, inflammation, oxidative stress, aging and estrogen signaling showing different patterns of activation between female and male ε4-positive brains (Hsu et al., 2019). One comparative study found that the ε4 isoform activates transcription factors like AP-1 more strongly than ε3 does in induced human-derived neurons and glia (Lin et al., 2018). ε4-associated AP-1 activation promotes APP transcription, leading to a rise in Aβ levels (Huang et al., 2017).

The presence of an *ε4* allele may also exacerbate disease pathogenesis and progression due to the absence of *ε2*- or *ε3-*associated neuroprotection. *APOE ε2* is more neuroprotective in women than in men, promoting survival and longevity (Rosvall et al., 2009; Schachter et al., 1994). Another study found that *APOE ε2* reduces LOAD risk in women more than in men (Neu et al., 2017); however, a more recent study did not confirm this (Reiman et al., 2020). *APOE ε3* may also be more protective against neurodegeneration in women, as this allele reduces total cerebrospinal fluid tau levels in women more than in men (Damoiseaux et al., 2012). In men, APOE ε4 exacerbates the contribution of andropause to increased LOAD risk (Panizzon et al., 2010). However, as andropause is more gradual than menopause, females could be more susceptible to the detrimental effects of ε4 earlier than men (Feldman et al., 2002; Harlow et al., 2012; Muller et al., 2003). In addition, accumulating evidence suggests that *APOE* variants drive sex-specific vulnerability to neurodegenerative diseases other than LOAD (reviewed in Box 2).

## Sex-by-gene interactions in LOAD

Genetic factors can differentially affect males and females; thus, when testing for genetic associations, it is crucial not only to include sex as a confounding factor but also to stratify association analyses by sex. The importance of sex and the sex stratification approach has been exemplified numerous times, and has enabled the identification of novel genetic risk loci for neurological diseases including not only LOAD, but also PD, ALS, MS and DLB (Table 1). As the use of polygenic risk scores (Box 1) is proving beneficial for predicting disease risk (Box 4), the same approach may be applied to sex-specific risk. Furthermore, family-based analytical designs, which robustly recapitulate population structure, can be employed to test for sex-specific disease risk loci (Prokopenko et al., 2020). In addition to the sex-dependent effect described for *APOE*, other genetic and genomic factors contribute to LOAD in a sex-specific manner, as we review here.

**Box 4. Polygenic risk scores for LOAD**

• A polygenic risk score (PRS) is a method of calculating disease risk by combining multiple disease-associated genetic variants in a single algorithm. While single subthreshold variants contribute little to disease risk, the performance of a combined risk score constructed from the weighted genome-wide association study (GWAS) single-nucleotide polymorphism (SNP) is much greater (area under the curve, 84%) (Escott-Price et al., 2017).

• PRS calculations were proposed based on thousands of LOAD-associated SNPs that do not reach genome-wide significance (Kunkle et al., 2019; Marioni et al., 2018).

• The potential advantages of PRS include:

(1) Enhanced predictability for LOAD diagnosis early in life

(2) Identification of convergent molecular pathways for the development of new therapeutic strategies and/or models in which to test therapies

(3) Selection of individuals with high PRS for clinical trials and precision medicine

(4) Enhanced understanding of early disease pathogenesis for biomarker development

• The PRS method is a powerful tool to predict the conversion from mild cognitive impairment to LOAD (Chaudhury et al., 2019), and PRS can be correlated to clinical outcomes, molecular signatures, neuropathology and brain function (Tasaki et al., 2018; Xiao et al., 2017).

• Although the majority of the predictability in these models comes from the contribution of *APOE*-associated risk, PRSs have predictive utility even for ε4 and ε2-negative individuals (Escott-Price et al., 2019).

• As sex-specific genetic associations with LOAD become more evident (Table 1), sex-stratified approaches are now more commonly applied, leading to the discovery of sex-dependent polygenic effects on LOAD risk. For example, these approaches revealed that the previously identified GWAS loci *BIN1* and *MS4A6A* contribute more to LOAD progression in women than in men (Fan et al., 2020).

### LOAD genetic loci

Genetic loci other than *APOE* exhibit sex-specific effects on LOAD risk, some of which directly relate to sex hormone activity and maintenance. For example, *CYP19A1* encodes aromatase, which is involved in the conversion of androgens to estrogens. Aromatase may be neuroprotective by increasing local estrogen levels in injured neurons (Azcoitia et al., 2001). Therefore, loss-of-function mutations can have detrimental effects in the brain. Indeed, several polymorphisms in *CYP19A1* increase LOAD risk in females (Table 1). Aromatase levels are increased in post-menopausal women but are decreased in the LOAD brain (Ishunina et al., 2007), which, when combined with loss of estrogen, can exacerbate disease progression. The sex-specific effect of *CYP19A1* variation depends on epistasis with a polymorphism in the gene encoding the anti-inflammatory cytokine interleukin-10 (*IL10*), which further suggests an interaction between hormonal balance and neuroinflammation (Table 1). In addition, the male-specific effect of androgen receptor (*AR*) gene variants on LOAD risk (Table 1) suggests that compromising AR-mediated neuroprotective effects of hormones increases vulnerability to neurodegeneration (Hammond et al., 2001).

Another example is *ESR1*, which encodes the estrogen receptor ERα. In addition to its sex-specific effects on LOAD risk (Table 1), polymorphisms in this gene affect cognitive impairment (Kravitz et al., 2006; Yaffe et al., 2009), metabolic syndrome (Goulart et al., 2009), ovarian (Yang et al., 2010) and cardiovascular function (Lawlor et al., 2006), and cholesterol levels (Sowers et al., 2006) in a sex-specific manner, suggesting that estrogen signaling dysregulation leads to widespread detrimental effects throughout the body. Many novel LOAD-associated single-nucleotide polymorphisms (SNPs) were found to be female specific in a recent GWAS; however, these have yet to be replicated (Nazarian et al., 2019). Other genes with known sex-specific effects in LOAD are related to blood pressure regulation, neurotrophin (Box 1) signaling, DNA replication, cognition, and mitochondrial and neuronal function (Table 1).

### Transcriptomic and epigenomic profiles

Non-coding variants, epigenomic signatures and transcriptomic changes associated with LOAD provide clues for the role of gene dysregulation in LOAD etiology. Sex differences in LOAD were reported at the genome sequence level (non-coding SNPs) and also observed for other ‘omics’ attributes, in particular transcriptomic and epigenomic profiles. Collectively, these studies suggest that gene dysregulation may, at least in part, be the underpinning mechanism for sex-dependent vulnerability to LOAD. Most recently, Mathys et al. characterized sex-specific cell subtype responses to LOAD pathology in human brain tissue and found that oligodendrocyte transcriptional activity becomes upregulated only in males, while excitatory and inhibitory neuron genes become downregulated only in females (Mathys et al., 2019). As aging is the biggest risk factor for LOAD, a microarray study revealed a striking 90% overlap between the prefrontal cortex gene expression patterns of healthy middle-aged females and male LOAD patients (Sanfilippo et al., 2019), suggesting that the female brain's transcriptional response to aging predisposes it to the development of LOAD. Other transcriptomics studies reinforce the idea that sex hormones are centrally involved in female-specific susceptibility to LOAD. For example, estrogen upregulates many of the genes found to be downregulated in female LOAD (Ratnakumar et al., 2019), consistent with previous studies demonstrating estrogen-mediated neuroprotection via genomic pathways. In addition, a meta-analysis of transcriptomic studies of LOAD generated protein-protein interaction networks, which centered on estrogen and androgen receptors (Winkler and Fox, 2013). As APOE ε4 is known to act as a transcription factor, one study found a strong correlation between *APOE* expression in *ε4*-carrying females and expression of genes involved in hormone pathways (Hsu et al., 2019).

As LOAD is likely to be caused by a complex interplay between genetic and environmental factors, it is no surprise that there are well-established epigenomic signatures of LOAD. Growing evidence suggests that sex-specific epigenomic signatures may be upstream of the transcriptomic profiles discussed above. Sex influences epigenetic factors like DNA methylation (Boks et al., 2009; El-Maarri et al., 2007). While some studies have failed to detect a significant effect of sex on LOAD-associated DNA methylation (Di Francesco et al., 2015; Kobayashi et al., 2016), others have identified a handful of specific loci that exhibit different methylation levels in females versus males with LOAD. For example, a methylome analysis of human neurons revealed that a CpG island in the promoter region of the mitotic gene aurora kinase C (*AURKC*) is hypomethylated in LOAD, leading to upregulation of *AURKC* (Mano et al., 2017). This same CpG island is hypermethylated in females compared to males with LOAD, and it also becomes hypomethylated as the number of *APOE ε4* alleles increases (Mano et al., 2017). This suggests that the presence of the *ε4* allele in females increases susceptibility to LOAD in part due to *AURKC* upregulation. Another study found a weak influence of sex on *TREM2* methylation in the peripheral leukocytes of LOAD patients (Ozaki et al., 2017). In addition, the promoter of *GPR50*, a candidate risk gene for LOAD, is hypermethylated in LOAD females and hypomethylated in LOAD males compared to healthy controls (Chen et al., 2019). The *App* promoter is hypermethylated, and thus transcriptionally silenced, in female wild-type mice (Mani and Thakur, 2006). However, the same study showed that *App* expression increased with ovariectomy and decreased with estradiol treatment (Mani and Thakur, 2006), suggesting that estrogen acts through genomic pathways to silence *App*.

Histone acetylation represents another layer of epigenomic regulation, and is hypothesized to play a role in LOAD pathogenesis (Graff et al., 2012). One study examined histone deacetylase activity in human brain tissue, specifically in cholinergic neurons containing pathological tau, and found that the abundance of histone deacetylase 2 (HDAC2) was reduced in females regardless of clinical diagnosis (normal, MCI or LOAD) (Mahady et al., 2019). As HDAC2 is involved in synaptic plasticity (Akhtar et al., 2009), neuroinflammation (Hsing et al., 2015) and cellular stress (Peng et al., 2015), gene dysregulation due to its reduction may be a contributing factor to neurodegeneration in the female brain. In addition, long non-coding (lnc)RNA regulation has been linked to the development of LOAD (Magistri et al., 2015; Zhou et al., 2019; Zhou and Xu, 2015); however, the role of sex in this relationship remained unclear until recently. A bioinformatics analysis of human LOAD brain microarray datasets revealed a total of 42 lncRNAs that were differentially expressed in LOAD versus normal tissue, 13 of which were sex specific, such as SLC25A25-AS1, PWRN1 and LY86-AS1 (Cao et al., 2019). FOXO signaling and glioma pathways were over-represented within the sex-specific lncRNAs (Cao et al., 2019). This work demonstrates that LOAD affects the expression of lncRNAs in a sex-dependent manner. Other types of non-coding RNAs may play a sex-specific role in LOAD pathogenesis; however, this was not confirmed in a recent study on microRNAs (Nelson et al., 2018). These findings highlight that integrative multi-omics studies will be essential to understanding whether epigenomic changes directly affect gene expression patterns in LOAD. This knowledge will be instrumental to further elucidating the molecular pathways underlying sex differences in LOAD susceptibility, as well as uncovering new therapeutic strategies.

## Findings from animal models

Several transgenic animal models harboring LOAD-related genetic modifications exhibit more severe disease-like phenotypes in females than males (Schmid et al., 2019; Wang et al., 2003; Yue et al., 2011). Although female rodents do not undergo menopause, they do experience a period of altered estrous cycling (Guimarães et al., 2015) followed by persistently low estrogen levels at old ages (Felicio et al., 1984), similar to peri- and post-menopause in women. Findings from these models are in agreement with two possibilities in humans: (1) reduced estrogen exposure in adult females exacerbates disease progression, as in J20 mice (Box 1) (Broestl et al., 2018); and/or (2) the organizational effects of sex hormones during neurodevelopment have lasting effects that increase susceptibility to neurodegenerative disease later in life, as in 3xTgAD mice (Box 1) (Carroll et al., 2010). For more information, we direct the reader to a review summarizing findings from LOAD animal models consistent and inconsistent with the sex differences observed in humans (Fisher et al., 2018). However, a more detailed examination of the interplay between APOE and hormones in rodent models can provide mechanistic insights for how these processes underlie sex differences in disease pathogenesis.

The detrimental effects of the *ε4* allele on APOE-estrogen interactions, centering on synaptic dysfunction and cellular stress, have been demonstrated in several rodent models. *APOE*-TR mice, in which the murine *Apoe* gene is replaced with human *APOE ε2*, *ε3* or *ε4*, have been used extensively in the study of molecular mechanisms of LOAD (Knouff et al., 1999). Following previous studies demonstrating that *APOE*-TR female mice with the *APOE ε4* genotype are more susceptible to cognitive dysfunction than males, one group demonstrated that N-methyl-D-aspartate (NMDA) receptor (NMDAR; Box 1) activation, linked to downstream effectors such as CaMKII (CAMK2A), ERK1/2 (MAPK3/MAPK1) and CREB proteins, is reduced in aged female *APOE*-TR *ε4* but not *ε3* mice (Yong et al., 2014). Deficiency in these NMDAR-related pathways may underlie the susceptibility of *ε4-*positive women to cognitive dysfunction via loss of neuroprotection and synaptic function. Similar to deficits in synaptic density found in human females, synaptic proteins are downregulated in *APOE*-TR *ε4* female mice, owing to dysregulation of factors involved in mitochondrial function and oxidative stress such as prohibitin 2, VDAC2, NADH dehydrogenase and GSTM1 (Shi et al., 2014). Also, like in humans, carrying the *ε4* genotype reduces APOE levels in *APOE*-TR mice (Yong et al., 2014), which may be mediated by decreases in 17β-hydroxysteroid dehydrogenase 10 (HSD17B10), a key enzyme in sex hormone synthesis, coupled with increases in aromatase (Shi et al., 2014). A recent study used *APOE*-TR mice to examine the interactive effects of sex, *APOE* and age on brain transcriptomic profiles, and found that these variables interactively influenced the expression of genes in the unfolded protein response pathway (Zhao et al., 2020b), a response to endoplasmic reticulum stress implicated in the etiology of neurodegenerative diseases (Scheper and Hoozemans, 2015). Together, these findings suggest that estrogen signaling is defective in *APOE ε4* carriers, and that loss of estrogen results in energy deficiencies and elevated cellular stress, particularly within synapses (Fig. 2). Reduced levels of and sensitivity to estrogen, exacerbated by both the *APOE ε4* allele and menopause, likely makes older women particularly vulnerable to neurodegenerative disease.

Animal models of AD also support hypotheses related to glucose hypometabolism and female susceptibility to LOAD. Compared to non-transgenic mice, aging female 3xTgAD mice show an exaggerated decline in expression and activity of transporters and enzymes needed for glucose metabolism, switching to ketone bodies as an alternative fuel in response (Ding et al., 2013a). Ovariectomy can induce this ketogenic switch in 3xTgAD mice, which is associated with increased expression of enzymes required for conversion of ketone bodies to acetyl-CoA (Ding et al., 2013b). In a rat model of perimenopause, a similar ketogenic switch was found to be regulated by insulin-like growth factor 1 (IGF1) and AMPK (PRKAA2)/PGC-1α signaling, as well as by ERβ expression (Yin et al., 2015). As ketone bodies are primarily derived from white matter, white matter volume, myelinated fiber volume and myelin sheath volume were all reduced in aged female APP/PS1 double-transgenic mice compared to males (Zhou et al., 2018). A recent metabolomic and transcriptomic study in *APOE*-TR mice confirmed that metabolic shifts in the female *ε4* brain are accompanied by loss of myelin integrity (Shang et al., 2020).

The ongoing progress in rodent modeling is expanding on what we have learned from ovariectomy models, which more closely recapitulate surgical rather than natural menopause. The recently developed accelerated ovarian failure model mimics natural menopause, as reviewed separately (Marongiu, 2019). Parallel studies of multiple models of different neurodegenerative diseases will also prove useful in identifying shared disease etiologies. For instance, a recent study highlighted a potential APOE-centered unifying mechanism in microglia underlying LOAD, ALS and MS across three animal models (Krasemann et al., 2017). As TREM2 is a putative receptor for APOE (Yeh et al., 2016), Krasemann and colleagues report that a TREM2-APOE pathway triggers a phenotypic switch in microglia from homeostatic to inflammatory through the microRNA miR-155 (Krasemann et al., 2017) (Fig. 2). Whether or not this pathway is influenced by sex hormones or different APOE isoforms remains to be studied.

## Conclusions

Here, we review evidence supporting three non-mutually exclusive hypotheses explaining sex-specific vulnerability to neurodegenerative diseases: (1) organizational effects of sex hormones during development; (2) synergistic effects of coexisting vascular, metabolic and inflammatory conditions that are risk factors for neurodegenerative diseases; and (3) irregularity and/or decline of sex hormone levels with aging. APOE is a multifunctional protein and therefore its role in LOAD pathogenesis is complex. However, it is becoming increasingly clear that estrogen is a key player in APOE ε4-mediated female-specific vulnerability to LOAD. The major mechanisms underlying this ε4-estrogen relationship seem to be negation of estrogen-associated neuroprotection and menopause-associated brain hypometabolism. Both human and animal studies support the overarching hypothesis that a ketogenic switch occurs as a response to the hypometabolic state triggered during perimenopause. Because ε4 can lengthen this transition period by delaying post-menopause, energy imbalance might represent the first step in ε4-associated toxicity in the female brain. Other important considerations in understanding the sex-specific effects of APOE ε4 include gene dysregulation via transcriptional effects and loss of female-specific protection conferred by the *ε2* or *ε3* alleles. Finally, animal models have largely aligned with studies in humans and have extended those findings to uncover potential causal mechanisms. Importantly, rodent models have revealed that estrogen signaling is defective in APOE ε4-positive individuals, leading to energy imbalance, cellular stress and synaptic dysfunction. All of this evidence highlights the need to carefully examine the role of sex in LOAD risk and, specifically, to test the hypotheses outlined herein. LOAD studies stratified by sex, such as integrative multi-omics profiling of disease-relevant human brain tissues, will enable the discovery of new genetic factors that remained masked in a sex-mixed sample and reveal known loci with greater effects in females or males. To achieve sufficient statistical power in studies stratified by sex, researchers now have access to more widespread and affordable whole-genome sequencing technologies than in previous analyses (Bansal and Boucher, 2019; Lightbody et al., 2019). This type of work will be essential for implementation in precision medicine based on sex, including the development of sex-specific diagnostic tools for clinical testing and identification of sex-specific targets for drug discovery in LOAD and other neurodegenerative diseases. In conclusion, this Review advocates for the importance of gene-by-sex interactions in studying the underpinning genetics of neurodegeneration, as many aspects of central nervous system function differ between males and females.
